# Towards a Moral Ecology of Pharmacological Cognitive Enhancement in British Universities

**DOI:** 10.1007/s12152-017-9336-5

**Published:** 2017-07-06

**Authors:** Meghana Kasturi Vagwala, Aude Bicquelet, Gabija Didziokaite, Ross Coomber, Oonagh Corrigan, Ilina Singh

**Affiliations:** 1 Trinity College of Arts and Sciences, Duke University, P.O. Box 95790, Duke University West Campus, Durham, NC 27708 USA; 2 NatCen Social Research, 35 Northampton Square, London, EC1V 0AX UK; 3 Department of Social Sciences, Loughborough University, Brockington Building, Margaret Keay Rd, Loughborough, LE11 3TU UK; 4 Griffith Criminology Institute, Mt Gravatt Campus, M10_4.02N, Brisbane, QLD 4122 Australia; 5 University of East Anglia, Norwich Research Park, Norwich, NR4 7TJ UK; 6 Department of Psychiatry and Oxford Uehiro Centre, University of Oxford, Warneford Ln, Oxford, OX3 7JX UK

**Keywords:** Ritalin, Adderall, Modafinil, Cognitive enhancement, Ethics, Smart drugs

## Abstract

Few empirical studies in the UK have examined the complex social patterns and values behind quantitative estimates of the prevalence of pharmacological cognitive enhancement (PCE). We conducted a qualitative investigation of the social dynamics and moral attitudes that shape PCE practices among university students in two major metropolitan areas in the UK. Our thematic analysis of eight focus groups (*n* = 66) suggests a moral ecology that operates within the social infrastructure of the university. We find that PCE resilience among UK university students is mediated by normative and cultural judgments disfavoring competitiveness and prescription drug taking. PCE risk can be augmented by social factors such as soft peer pressure and normalization of enhancement within social and institutional networks. We suggest that moral ecological dynamics should be viewed as key mechanisms of PCE risk and resilience in universities. Effective PCE governance within universities should therefore attend to developing further understanding of the moral ecologies of PCE.

## Background

From its nascence in the early twenty-first century, the field of neuroethics has been tightly tied to the issue of cognitive enhancement [1]. Though bioethicists have grappled with the treatment-enhancement debate for decades, in the case of cognitive enhancement, the vast array of mental states that are considered “normal,” and the largely uncharted nature of the human brain blur this classical dichotomy [2]. Out of this ambiguity arise several important ethical questions, which have been addressed from a number of disciplinary perspectives: How do we recognize and measure enhancements in cognition [3]? What are the impacts of cognitive enhancement on the moral and social development of the individual [4]? What are the effects of pharmacological cognitive enhancement (PCE), the most accessible means of cognitive enhancement, for communities, institutions, and societies [5–7]?

Descriptive ethics, the study of people’s beliefs about morality [8], lies at the intersection of social scientific and philosophical approaches to the study of cognitive enhancement. This approach is best oriented to understanding *attitudes and practices* around PCE, thereby supplementing surveys of PCE prevalence with richly detailed and actionable information. The results of descriptive ethical work can also be incorporated into normative ethical arguments, which then bolster the feasibility of theory and of policy recommendations [9]. Recent scholarship in moral philosophy has underscored the importance of empirical contributions to ethics, and has highlighted descriptive ethics as “a topic unduly neglected by contemporary philosophers” [10].

There is a critical knowledge gap in systematic and evidence-based understanding PCE among British university students. A non-representative survey among British university students found relatively low lifetime prevalence rates for the three most commonly used pharmacological cognitive enhancers: 6.2% for modafinil, 5.9% for methylphenidate (e.g. Ritalin), and 2% for Adderall [11]. The majority of students had engaged in PCE opportunistically. The percentage of on-going users of PCEs was approximately 1% for Ritalin and Adderall, and approximately 3% for the most accessible drug, modafinil. Similarly, a population survey conducted by the Wellcome Trust charity put on-going use of ‘cognitive enhancement drugs’ in the general population at 2% [12]. The findings of these surveys contrast sharply with surveys conducted by newspapers in elite UK universities; e.g. the Cambridge Varsity and the Oxford Tab [13, 14]. In these oft-cited surveys, overall prevalence is reported as between 15 and 18%, though variations among different colleges within the universities are noted.

Even less is known about the factors that shape PCE practices among British university students. To date, only one qualitative research project has been published, based on a sample of 13 British university students who were all modafinil users [15]. Researchers found that modafinil was acquired either via the Internet or through friends, and used to “catch up” to high achieving students. Similarly, there is little research-led understanding of the factors that shape UK university students’ PCE practices. Several UK studies suggest that a majority of respondents do not find PCE inherently unethical [16, 17]. At the same time, the most accessible drug in the UK, modafinil, is used consistently by a very small minority of students. To better understand the factors that shape PCE practices, some leading PCE researchers emphasise the need for “sociocultural research strategies” that examine “pharmacological enhancement as a broader phenomenon…and conside[r] the individual in his/her cultural, social and legislative environment.” As one set of authors has argued, studies need to take account of “country-specific cultural and regulatory factors that may influence the extent of cognitive enhancement and attitudes towards it” ([18], p. 275). Framing PCE as a broader socio-cultural phenomenon also highlights the need for research among PCE non-users as well as users [5, 7, 15, 19].

Thus, there is a desideratum for a descriptive ethics approach to studying PCE among British university students that takes into account not only individual attitudes and experiences relating to PCE, but also students’ social environments, the university context, and overarching national trends. Research conducted in other countries suggests that a range of social dynamics; such as peer-pressure, competition, performance demands, and prior drug use, are highly influential in shaping PCE practices in universities [4, 20–24]. Social dynamics are defined as the processes by which “individuals are directly influenced by the choices and characteristics of others, creating a feedback loop from the past choices of some people to the current social context and future choices of others” [25]. Social groups also have the power to encourage PCE through “soft peer pressure,” defined as “an implicit social endorsement in which one has specific knowledge that peers are enhancing, albeit no explicit pressure from peers to join in” [20]. Students worry disproportionately about the coercive influence of peers to try PCE. Faulmüller, Maslen, and Sio’s comprehensive literature review on attitudes towards cognitive enhancement found that coercion is one of study participants’ top three concerns [26].

However, social dynamics could also discourage PCE. In a discussion of social networks, non-using peers of users were found to often harbour negative opinions of PCE and suggested that stigma discourages use or, at the least, the admission of drug use [26]. Both in the explicit form of coercion, and in the implicit form of soft peer pressure, these influences can pose a threat to autonomous decision-making in relation to PCE, which users and non-users alike emphasise as a key value in the ethics of PCE [20, 27, 28]. Other factors that discourage PCE include safety concerns about serious side effects, policy regulations that “activate social norms against usage,” and costly sanctions that discourage use [9, 23].

In addition to supplying a dose of peer pressure, individual acquaintances, friends, and social groups can also provide direct access to PCE and normalize [29]. PCE as part of a group’s culture. Group PCE practices have been documented most extensively in American fraternities, where PCE prevalence estimates are as high as 55%. [30, 31]. Fraternities are tight-knit groups on campus, known to support each other socially and academically. While fraternities are not prevalent on UK campuses, the studies shed light on the role of university group cultures in PCE practices. Relatedly, Sattler identified the “contagion effect,” an individual’s higher willingness to use cognitive enhancement drugs when there are more individuals who use them in the social network [23].

Competition is another key sociocultural factor driving cognitive enhancement. PCE prevalence estimates are higher in the American northeast, the hub for the country’s most selective universities [32, 33]. An analysis of over 200,000 tweets containing the word “Adderall” found that these tweets were concentrated geographically in the American northeast and temporally during the time of final examinations in universities [34]. Socioeconomic background also plays a role in inspiring a competitive spirit: students from high-income bracket families have reported shouldering more expectations to achieve and have been identified as at a higher risk for PCE [35]. In a qualitative study of life-context of pharmacological academic performance enhancement among university students, German researchers observed competition as motivation for PCE [19]. As one student put it, “I want to be better than my peer group and fellow students, although I realize that that sounds rather anti-social.” The role of competition and the value of competitiveness in relation to PCE practices has not been examined systematically in the British context to date. The divergence in anecdotal PCE prevalence reports, between the UK’s elite collegiate universities and other British universities, suggests that this is an important area to understand.

The study described in this paper is the largest (*n* = 66) qualitative study of PCE among students in British universities to date. We deployed focus group methodology to provide an interactive space for students to express views and to share experiences about PCE. We also analysed focus group interactions themselves to gain further insights on the social dynamics around PCE. This approach allowed us to understand how individual attitudes and practices interacted with broader social dynamics and cultural influences, which we organise drawing on the concept of a moral ecology [36]. Our findings suggest that effective governance of PCE must engage the moral ecologies of PCE in universities in order to effectively minimise PCE risks and to maximise any PCE goods.

## Methods

### Participants & Recruitment

Research ethics approval for this study was obtained from the research ethics committee at the London School of Economics and Political Science. All participants provided written consent. During the focus groups, participants were given a pseudonym, to ensure that they could stay anonymous in the group context, if they so chose. Participants completed an anonymous demographic questionnaire following the focus group session.

Participants were recruited purposively through a market research company and through word of mouth. To participate in the study, participants had to be enrolled on a degree course at a university in the United Kingdom. We held focus groups in the Greater London area and the South West of England area.

The total number of participants was 66; the majority were White students enrolled on an undergraduate degree course. Just over one third of students attended a Russell Group university.^1^ About 10% of students identified as non-White; gender was equally represented in the sample. Fifteen universities were represented across 8 focus groups.

### Data Collection and Analysis

Four moderators were involved in the conduct of the focus groups, each lasting a total of 2 h.

Focus groups are a qualitative, empirical method used to understand perceptions, experiences, practices and social dynamics [37, 38]. When carefully conducted, the focus group produces two data streams: group views; and the discursive dynamics of the group that give rise to knowledge claims and semantic ‘outcomes.’ In our focus groups, moderators followed a structured protocol to ensure that all focus groups held discussions on the same topics, in response to similarly worded prompts. The protocol (available upon request) included a set of open questions, responses to short scripted scenarios, and a group-sorting task.

Thematic Analysis (TA) was used to analyze the focus group transcripts [39, 40]. A wide variety of models and approaches have been developed by scholars using TA [39, 41–44]. We adopted a ‘classic’ approach, based on the following procedures:

1. Coders immerse themselves in the data
2. Data is read word by word to derive themes
3. A coding scheme is developed and tested on a sample of text
4. Coders check for potential coding disagreements
5. Once disagreements are resolved, the full corpus of text is coded
6. Important themes are then sorted into categories

Ideally, themes are generated inductively from the raw information or deductively from theory or prior research [44]; however, in practice, the elicitation of themes is often an abductive process, based on iterative cycles of coding. To capture group dynamics (communication processes, interactions, consensus/disagreements and the evolution of viewpoints), we supplemented our analysis of individual-level data with close examination of group interactions using a combination of coding and team discussions to focus our analysis.

## Results

After coding utterances of attitudes and practices relating to PCE, six main themes emerged from the focus group data. We organised these themes into three topographical levels:

- **Individual Level**

1. *Awareness of PCE:* Most individuals have few personal experiences with PCE, but can identify popular drugs
2. *Fairness and Safety*: Valuing autonomy promotes acceptance of PCE, while risk aversion promotes disapproval

- **Peer group Level**

3. *Access*: On & off-line social networks provide access to vendors and quality control information
4. *Soft Peer Pressure:* Social endorsements and discouragements of PCE influence individual choices

- **Social Environment Level**

5. *Contagion and Containment:* PCE is distributed and contained locally, with institutional and national cultures influencing prevalence
6. *Bias Against “American” Values:* Medicalization of academic competition is viewed as an undesirable American phenomenon

**Fig. 1:**
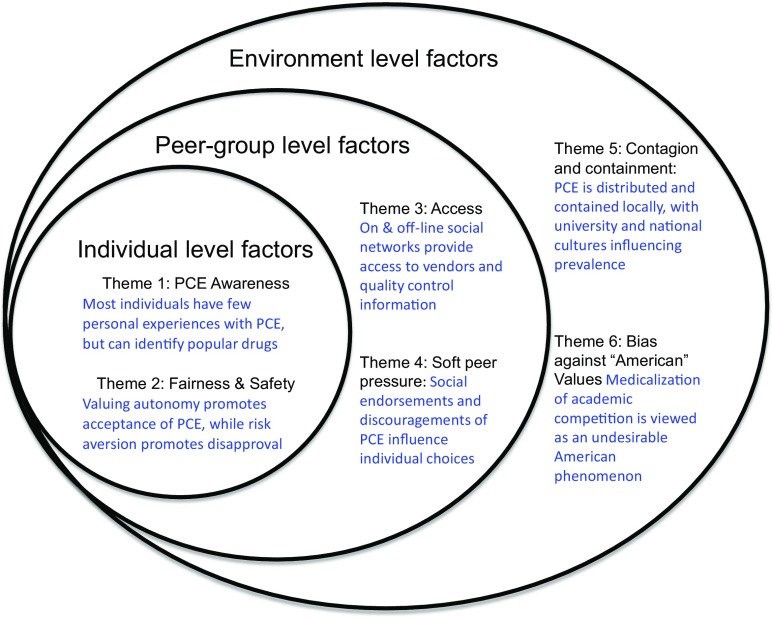
Higher-order dimensions of the theorized moral ecology around PCE subsume layers of social interactions that contain micro-level processes

The first two themes outlined below, “awareness of PCE” and “fairness and safety,” focus on the individual’s personal engagement with PCE and evaluation of consumption. The next two themes, “common ports of access” and “soft peer pressure” broaden to the social level, explaining the role of friendship groups in providing access and soft peer pressure, either to encourage or discourage PCE. The final two themes, “contagion and containment” and “bias against American PCE values”, broaden the scope to social and national environments that subsume peer groups and individuals, examining opinions on drug use and competition that students categorize as characteristic of highly competitive cultures (see Fig. 1).

In what follows, we use the standard convention *n* to represent the number of mentions, not unique speakers. Discussion among focus group members is illustrated by providing the transcribed comments of several speakers without interruption.

## Awareness of PCE

Thematic Summary: Most individuals have few personal experiences with PCE, but can identify popular kinds of PCE substances.

We tabulated 89 mentions of non-consumption and 36 mentions of consumption (not necessarily by the speaker) of PCE by focus group participants. The majority of our focus group participants did not report engagement with PCE. A significant minority of students did not know the names of any cognitive enhancement drugs. “I have not a clue about that sort of thing” was a typical response when members of this group were asked about PCE.

Among the group that could name PCE substances, Adderall, Ritalin, and modafinil were the drugs most commonly mentioned. Modafinil was mentioned less frequently than the former two. A discussion between two participants elaborates on changes in the PCE landscape within the past two years:

Calvin: Ritalin... I’ve never heard any experience of it being available. modafinil… it’s not freely available, but it’s sort of like... you’re finding it if you want it.

Anthony: Well, I haven’t been in the UK for two years because I was on a year abroad, so I don’t know, is that [the availability of modafinil] a recent thing?

Calvin: Yes, it is fairly recent I think in that I see word starting to spread about it, but it’s not… it’s not even close to being mainstream yet in any sort of way.

Timothy: I mean, I was aware of Ritalin from, like, media coverage of it for a few years, but then modafinil has been a much more recent thing, then the caffeine pills. I mean, it’s kind of available in shops and everywhere so I’ve been sort of aware of them for a while.

## Fairness and Safety

Thematic Summary: Valuing autonomy promotes acceptance of PCE, while risk aversion promotes disapproval.

In general, autonomy interests trumped concerns about unfairness of using PCE. When asked about PCE use during exams (school context), students were more likely to discuss reasons why PCE was morally acceptable than to discuss its unfairness. In the exam scenario, some students responded that PCE would be unfair (*n* = 50) and risky (*n* = 10). However, students also thought that PCE was a matter of free or personal choice (*n* = 58); or a good choice (*n* = 6); or a pragmatic choice (*n* = 12).

The discussion below captures this diversity of opinion about an exam scenario:

Moderator: It’s exam week, right, and you’re standing in the hallway looking, waiting to go in, and the person behind you, you see her swallow what you know to be a tablet of Ritalin, going into the exam. So, let’s just go around the room and see what your first reaction is.

Sue: All these drugs are doing is just speeding up the natural processes in your brain, so they have done nothing more than like the vitamin supplements that you take in the morning, so yes, I don’t have a problem with it.

Matthew: I think I’d have a problem with it because... as it stands Ritalin is, sort of… that person’s getting an unfair advantage. ...if you saw someone, I don’t know, with, like, a piece of paper with the answers on it, I’m sure everyone would be a bit peeved. So...like you were saying about it’s no different from vitamins and stuff, but it’s totally different because a vitamin supplement wouldn’t increase your concentration, because if it did then everyone would take it the same way because they’re legal.

Focus group participants were encouraged to reflect on their expressed attitudes to PCE in the school context by considering another scenario in which PCE use on an exam might have advantaged the user in an employment context. Here a majority of statements (*n* = 29) still promoted acceptance of PCE. The belief that PCE use in exams, that subsequently advantaged the user in an employment context, constituted cheating was also quite commonly expressed among respondents (*n* = 19). The following excerpt exemplifies the discussion about PCE in the employment context:

Moderator: Say that you are at a job interview, and you’re waiting with the same person that you saw pop the pill before the exam, and then later you find out that the person got the job. Would you feel any different?

Thomas: Yes, I’d be pretty pissed off.

Stephanie: And I’d hate her as well.

Moderator: So you would think worse of her?

Thomas: No, I think she’s probably cleverer taking the drug. If she knows it’s going to increase her concentration and you knew about it, you could have taken it, you had the option not to and you didn’t, but she’s taken it and she’s got the job. You were scared of the risks and she wasn’t, and it paid off

Moderator: But you’re saying something different, which is interesting, you’re saying not that she cheated so much, but that you were, sort of, the fool for not knowing.

Jean: It’s like she played the game better than anyone.

Outside these specific scenarios, the most common reasons given for acceptability of PCE was the right to individual autonomy (*n* = 48) and the pragmatic benefits (*n* = 21). The idea that PCE encouraged wrong values was expressed 39 times, with participants arguing that PCE was either intrinsically wrong, or promoted social dynamics that undermined collective relationships.

Students also expressed significant concerns about the risks of PCE. General reasons for not using PCEs covered concerns about risk about addiction (*n* = 53) and side effects (*n* = 25). A common justification for the idea that PCE is a “good choice” was the argument that engaging in PCE is the same as using other means to get an advantage on others, in order to reach a desired end. Students also argued that PCE was comparable to other forms of ‘enhancement’ (e.g. vitamins, cognitive training games), and was therefore morally acceptable. However, this pragmatism was not often paired with individual willingness to use PCE. Many who found PCE acceptable did not want to personally engage in it.

## Access to PCEs

Thematic Summary: On & off-line social networks provide access to vendors and quality control information

Students identified the Internet (*n* = 13), personal networks (*n* = 6), and ADHD prescriptions (*n* = 4) as the main ways in which they thought one could access PCE.

The Internet was regarded as a convenient means of accessing cognitive enhancement drugs. As one participant said, “You literally type it in [a search engine] and I’m sure the first thing is where to get it.” When asked about how they would assess the reliability of an Internet vendor, multiple students said that they would get recommendations from friends who had successfully purchased cognitive enhancement drugs in the past.

A very small minority of students in the focus groups had experience of accessing PCE online. Other students were interested in hearing about these experiences. One participant described using the (now closed) darknet website Silk Road to purchase modafinil:

Noah: Do you get these on the black market? Where do you get these drugs?

Jane: The black market of course.

Moderator: What is the black market?

Jane: The places and the people that you buy illegal drugs from. Internet people.

Noah: I always thought it would be hard to get on the internet for some reason.

Jane: No, no, no, no, very very easy. You just have to be a little bit tech savvy.

Moderator: What does that mean?

Jane: Sure, um, there’s one website called the Silk Road. Um, to access it you need to have a... uh, sort of, a programme on your computer called Tor...

Aside from the Internet, students accessed PCE via friends or family who had ADHD prescriptions or access otherwise. They described an open sharing ethos in which friends freely gave away their stimulants: “Some of my friends have ADHD and they are very generous with their medication and prescription.”

As with the social supply of recreational drugs, even when cognitive enhancement drugs were sold, they were distributed within friendship groups and for marginal profits:

Noah: Do you make money off these pills?

Jane: Not really. I just... I sell them to my friends for... and I make a profit of, like, 10p or something per day. So if I supply them for, like, seven days I make, like, 70p. Yes, I’m not, like, a drug dealer or anything.

## Soft Peer Pressure

Thematic Summary: Social endorsements and discouragements of PCE influence individual choices.

In addition to providing logistical access, peers can influence attitudes by encouraging or discouraging PCE. Participants reported soft, or indirect peer pressure to engage in PCE:

Michael: If I had a group of five friends and I was only one that wasn’t taking them then I'd feel more inclined to take it [inaudible].

Theresa: But there are some people, like I have another friend who would happily take anything just to get them through and it’s usually things like peer pressure and that. It’s just, you know, what sort of stage are you at where you’re prepared to take it in the first place? You could be under severe stress and strain and you feel like you’re lost, you have no way out and you feel that if you, you know, failure is like the last option and you’re going to take anything...

In friendship groups where PCE was not present, peer influence was seen to discourage use. When asked about the social acceptability of PCE and how they would feel about admitting to engaging in PCE, the majority of participants said that they would not feel confident making an admission to their personal networks. A main factor in this lack of confidence was perceived stigma, which was driven by perceptions of PCE as socially unacceptable in mainstream British culture.

Moderator: Okay, and ...if you used cognitive enhancers do you think you’d ... tell your friends about it?

Amber: I’d be embarrassed.

Kurt: And I wouldn’t want them to think I’m cheating if I took it in the first place.

Amy: I think as long as it’s still quite unknown, like it is in England, like relatively, I don’t think I would want to because just like how we all reacted, everyone would, kind of... I don’t know.

Rick: If my friend’s probably a drug user then I’d probably be like yes, I think you should take this, but if it was another [unclear] who was completely against drugs, I don’t fancy telling him about taking Ritalin because he’d just look completely down on you.

Hannah: I don’t reckon this will hit off in England. I don’t know, like amongst my friends at uni, if I turned around tomorrow and said oh yes, I’m taking these cognitive drugs, I think they’d be quite snotty about it.

## Contagion and Containment

Thematic Summary: PCE is distributed and contained locally, with institutional and national cultures influencing prevalence.

Within the discussion of the limits of PCE, participants noted that PCE might be a socially boundaried phenomenon: use was low in the general campus population, but high in certain social groups. Students commented that, given the diversity of the university population, it was probable that there existed friendship groups who engaged in PCE, even if the “vast majority” of the university population did not use them.

Participants elaborated on the pattern of concentrated use within friendship groups:

Connor: It was fairly widespread amongst one friend group, but then outside of that friend group it would be non-existent. Like, people would have heard of it but they wouldn’t be... like almost none actually. And so the reason it was in that friends group is one person found it and then said, oh, you should try this, and then through word spreading it became quite common through that. Like if you speak to someone outside that friend group and outside university, then they wouldn’t ...know what you were talking about in any real sense, I don’t think.

The university was seen by many as an institutional breeding ground for PCE. One participant delineated factors that promoted PCE in universities, and contrasted these with conditions at his secondary school:

Peter: School, you, like, are kind of trying to achieve something as a group but at university you probably left your home, your solid friends and you come into it on the same footing as everyone else and you’re just going to go out of it, you know, it’s more about what you take from university than what they give you, so.

Within universities, participants were keenly aware that highly ranked institutions were likely to constitute an environment in which PCE was more prevalent:

Adam: Through my friends who were at LSE [London School of Economics] and also at Oxford or Cambridge... I get the idea that it’s much more prevalent amongst higher ranking universities - maybe where there’s more pressure than at, say… at the lower end. But I think maybe because that pressure is not there, the workload is not quite the same; it’s not as prevalent.

Uma: I’d agree with what Adam said, the only experience that I’d had hearing about it was when my friend went to Harvard and some people there were using it … they were described to me as kind of not the smartest kids in the class but the most competitive so they wanted a bit of an edge.

The association of PCE with the academic and social elite was a recurring motif:

Brian: I can actually see parents giving their kids drugs like this because, I mean, if you want your child to be a doctor at Cambridge or something, and you want them to sit down and do their homework, I’m pretty sure you can find something…

The role of parents in creating an environment that normalized PCE was also common. Several participants mentioned knowing someone whose parents were physicians and prescribed ADHD medications for their children without a diagnosis. One participant described a link between parenting and university context:

“Marcus: I’d say it [levels of PCE] definitely depends on the university, the type of people that go to the university. Like you said, I’d say it does appeal to two ends of the spectrum; the people that cram things last minute, I need to do what I can to pass this, or there are the people whose parents are very, very pushy...And if you get a university that’s, well any institution that’s got those two groups in large numbers, it’s going to have that effect.”

A broader reported social influence on PCE was national ‘drug cultures’. These drug cultures, as defined by focus group participants, were broadly related to recreational drugs on the one hand, and to prescription drugs on the other. A ‘drug culture’ was reportedly constituted of local (peer and university) as well as national attitudes and social policies in relation to drugs. Participants consistently raised arguments that generalized differences between drug cultures in America and UK. In a typical account, a participant identified an American “drug culture,” in which use and overuse of drugs in day-to-day life is normalized.

Lauren: My friend, she’s got extremely rich friends who live in Santa Barbara and all the kids are just whacked on drugs the whole time, and that’s not an exaggeration. All of them are on coke, everything, and their parents, everything, like it’s completely mental. And she says it’s like it’s a massive problem there but it’s not that talked about. And I think that must just, if the rich kids are doing it then it spreads into universities and then everyone else starts doing it

In the focus group discussion, students accepted the Santa Barbara example as standard American behavior. Participants referred to this perceived normalization of drugs when explaining perceived differences in PCE prevalence between the two countries. America was seen to harbor the sort of “pill popping” culture in which PCE was not given much forethought because of widespread use of diverse drugs and ease of access.

## Bias against “American” Values:

Thematic summary: Medicalization and academic competition are viewed as undesirable and American phenomena

Focus group participants expressed a common view of America as a culture that encouraged medical labels and medical products as solutions to everyday problems:

Thomas: I mean, I’m biased against Americans - I’m just saying it because it’s true - I think they’re much more sort of naïve, you know, are sort of, oh, my head hurts, I’ll take a pill, like cause and effects, directly you know solve one problem not thinking about everything else that, you know, Aspirin does like thin your blood or I don’t know what, things like that.

Students were also highly critical of practices that allowed prescriptions to be obtained for “... [a]ny kind of attention disorder or any kind of abnormal behaviour... the solution for that is always give them drugs, give them drugs, give them drugs”. The social acceptance of prescription drug use was seen to provide easy access to stimulants, which could be used for cognitive enhancement.

Participants linked medicalization to permissive pharmaceutical advertising policies, which were seen as distinctively American:

John: I think in America it’s a bit different though because they’re so big into their advertising and I think they sort of believe everything they read in a way. They’re very easy to scam. But that’s just my opinion.

Leah: I don’t think any of us would do it because none of us are American.

The notion of PCE itself being an American phenomenon was commonly expressed (*n* = 40), but there was some resistance to a stereotypical portrayal of American prescription drug culture. Some participants suggested that PCE was not ‘American’ so much as something culturally ‘different’:

Amy: I think it probably is more American but only because I just can’t imagine.... I don’t know anyone who takes it. It’s just, like, when I saw it on 90210... so that’s why I think it’s probably more American but only because it seems more alien to me.

Mary: I wouldn’t even say it’s really American... they’re quick to dish out legal drugs more than over here, I think. But in terms of illegal ones, I don’t know…

The value of competition also shaped perceptions of PCE. We coded four reasons focus group participants said they used or thought their peers engaged in PCE: competitive advantage (*n* = 31), curiosity (*n* = 11), opportunity (*n* = 18), and pragmatism (*n* = 48). Of these, the most common justification for PCE was pragmatism: students thought that PCE achieved instrumental outcomes, such as making a necessary task more feasible. Instrumental outcomes were contrasted with enhancement outcomes, which would achieve superiority or a competitive edge:

Blaire: I know people can work longer and for most people I don’t think it’s about, like, being super and, like, learning more -- it’s just if you need to learn X amount in however many days.

Moderator: Yes, then you use this as a kind of a tool to get you there.

Blaire: Yes.

Participants expressed a common view that competition was the primary driver of PCE in America. The distinction between American competitiveness and British pragmatism was claimed explicitly and implicitly; for example, it was not uncommon for participants to emphasise, like Blaire, that British PCE users would not have an interest in “being super.” While participants admitted that their perceptions might be grounded in stereotypes about Americans, their opinions converged on an image of competitive, pressurised American students who engaged in PCE at high rates to get ahead. British students, on the other hand, were framed as relaxed, skeptical and pragmatic in their approach to PCE.

These portraits of individual cognitive enhancement drug users in America and the UK were further supported by perceptions of different societal values in the two countries. Participants echoed the opinion that America was overall a “more competitive society,” than the UK (*n* = 4), and that universities in particular draw forth a spirit of competition:

Moderator: Does [engaging in PCE] sound like an American thing to you guys?

Theresa: Yes.

Rick: Yes, it does. Yes.

Michael: Yes, it does sound quite American.

Moderator: And what about it makes it seem more American than British?

Michael: I think it’s because it’s quite a quick solution and that’s what you kind of associate America with is speed and everything going really fast. Yes, that’s like Americanisation, really, isn’t it?

Competitiveness in American life was seen to be a value iteratively reinforced in families and universities; focus group participants thought that American students were “all doing their best for their parents because they've got to do really well...ahead of everyone else.” Some participants framed competitiveness as an obligation to families, while others framed it more generally as part of a pressurised environment:

Sam: I think Americans are kind of more pressured into getting higher grades and stuff as well. They’re always going on about getting straight As and stuff so I think, in the UK education obviously is important but I don’t think they see it as important as the Americans do.

In contrast, coded nodes on the theme of competition in the focus group transcripts yielded a substantive characterization of British students as non-competitive. A typical statement was: “I just don’t see my degree as a competition with everyone else who’s doing it. That’s what I get for myself.” One student who self-identified as competitive, qualified his statement to make it clear that his competitiveness was self- rather than other-directed: “I’m more like competitive on a personal level, like trying to outdo myself constantly.”

## Discussion

### Towards a Moral Ecology of Pharmacological Cognitive Enhancement

The topography of the social dynamics around PCE is multi-layered. It includes the factual knowledge, moral attitudes, and safety concerns of the individual. The individual is situated within peer groups, which facilitate access and social influence, creating a tension between the individual’s desire for autonomy and both explicit and implicit pressures from peers. These peer groups operate within the university environment, in which social dynamics are influenced by both institutional and national cultures. These influences subsequently modulate levels of competition within the environment. Finally, values and regulations at a national level have trickle-down effects on the layers they subsume.

Our analysis suggests that factors at individual, peer-group and environmental levels interact to influence moral attitudes to PCE that have practical effects on PCE practices within British universities. To capture these multi-level dynamics in the university setting, we draw on the concept of a ‘moral ecology’: described as a framework that captures the relationship between human dynamics, individual morality, and social systems [36]. The moral ecology positions the university as an ecological niche in which cognitive enhancement drugs are situated. Within the niche, individual, group and collective dynamics interact to foster moral attitudes and practices that are seen to promote the balance required for general flourishing. The ecological analogy also emphasises the complexity of the multi-level system that is the modern university; an account that focuses on only one level of activity within this system, such as individual decision-making, is necessarily limited in perspective and explanatory power. It is possible to identify key moral forces within the system, and to illustrate their impact across the system, as we illustrate below.

### Peer Influences on PCE in UK Universities

Around the world, quantitative reports of PCE prevalence among university students demonstrate that PCE use is the exception, not the norm [11, 33, 35, 45, 46]. Our focus group study of university students based in two major urban areas in England found low use of, and low knowledge about PCE. This finding contrasts with media representations of PCE in the UK: A thematic content analysis of 142 newspaper articles found that 94% of articles portrayed cognitive enhancement as common, increasing, or both [6].

The media message that PCE is both widespread and rapidly growing could contribute to false beliefs among students, with deleterious consequences: students who mistakenly believe that the majority of their peers use drugs are more likely to misuse both prescription stimulants and illicit drugs [47, 48]. An increase in the overall number of students who misuse drugs can influence both attitudes and practices: A major predictor for PCE is awareness of other students engaging in PCE [11, 28, 23]. However, our findings suggest that the impact of these broad social influences is mediated at the peer group level. In the focus groups, PCE was perceived to be a socially bounded phenomenon, with peer influence on use thought to be stronger within user groups, but less influential outside those groups. Still, students on the outside knew which groups were likely to have direct access to cognitive enhancement drugs; and they also knew which of their peers would be able to advise on the best route to internet access of means of PCE.

In general, the focus groups illustrated that PCE in the university setting is largely built upon social networks and social sharing. Sharing and other forms of non- commercially orientated supply of recreational drugs among friends and acquaintances is widely known as ‘social supply’ [49], and is common in drug-using groups where the drug involved is relatively normalised. Peers who provided PCE to others did not see themselves as, or identify with the label of, a ‘dealer’. This is common among social suppliers and may reflect a need to justify social supply in morally neutral terms [50].

In the context of PCE, coercion was reported to be more subtle and indirect than the peer pressure classically associated with alcohol or cigarette consumption [51, 52]. Our focus group participants reported no incidents in which they felt forcefully encouraged to use cognitive enhancement drugs. Instead, coercive forces took the form of soft peer pressure, in which students are exposed to these drugs and start taking them in an experimental way. Friendship groups facilitate the transmission of PCE, providing what we call ‘pockets of access’ that encourage PCE. As at least one other focus group study has shown, students who are situated within such pockets of access articulate a tension between a belief that PCE should be an autonomous, voluntary decision and the reality that certain social environments motivate and encourage PCE [28].

It will be important to identify and examine such pockets of access in order to understand the potential social and individual harms, and the potential benefits of PCE in universities. As outlined in the introduction, American research shows that soft peer pressure operates within university fraternities and sororities in particular [31]. In the UK, we might expect to find similar pockets of access within different courses of study, or across colleges within a collegiate university. Examination of the social dynamics in these pockets of access within university settings is also likely to provide better understanding of the circulation of cognitive enhancement drugs. It could also be illuminating to explore attitudes towards PCE in relation to the particular programmes of study students are undertaking. For example, we noted that some medical students reported low engagement with PCE, but they were knowledgeable about cognitive enhancing drugs and familiar with using high caffeine drinks to enhance cognitive function. Given that medical school environments in the UK are highly competitive [53] and medical students experience high levels of stress [54], it may be that these factors create the kind of environment for the social acceptability of PCE. Further research on these issues will enable development of more precise avenues for management of harms related to PCE engagement.

### Collective Values Moderate UK Students’ Engagement with PCE

Our findings suggest that a majority of British students have strong moral attitudes to the value of competition and to use of prescription drugs for cognitive enhancement. Students in our focus groups also specifically contrasted a British attitude to competition and prescription drugs with an American attitude. These contrasts served both as explanations for low levels of PCE in the UK and as moral reasons why British students should not adopt perceived American practices. The contrast between British and American student values is supported by some other studies: sociological research among British students finds that they assign less importance to the individualistic values of achievement, hedonism, self-direction, and stimulation than American students [55]. However, it is also notable that studies suggest British students use non-prescription substances for cognitive enhancement at relatively high levels; a majority of students in a national survey said they had used caffeine tablets for cognitive enhancement [11] and students in the focus groups commonly suggested that highly caffeinated energy drinks and caffeine tablets such as Pro Plus were ‘every day’ and extremely prevalent. Students speak openly about using these drinks and tablets; there is low associated social stigma, and the practice is not viewed as indicating individual competitiveness. Further research is needed to understand why British students draw a line of acceptability between cognitive enhancers that are freely available non- pharmacological substances and those that are prescription drugs.

Values around competition and stereotypes about American competitiveness and drug-taking have practical moral importance in the social dynamics of PCE in UK universities. Such values are less likely to be expressed in larger-scale investigations of PCE, such as surveys, because they refer to a set of implicit normative anchors that give shape to social behaviours within academic cultures. Focus groups can be seen to represent a microcosm of social dynamics around PCE in UK universities; therefore the potentially biasing effects of focus group dynamics themselves should be noted. In our focus groups, competitiveness was identified by a majority of participants as an “Americanism.” Participants who self- identified as competitive might have been silenced by social desirability bias, unable to make an admission of competitiveness to a group of peers who viewed it as a stereotypically negative and American quality. It is possible that this silencing dynamic operates at a more general level within British universities, to keep engagement with PCE low.

Perceptions of competitiveness within American culture in general, and American universities in particular, might also have been exaggerated in focus group discussions, much as they are in general societal discourse. The accuracy of a moral belief does not necessarily impinge upon the strength of that belief, and stereotypes are particularly difficult to shift [56–58]. However, weakly evidenced beliefs are presumably more likely to be malleable when challenged, particularly in a pedagogical setting that values and promotes evidence-based arguments. The strength of competition as a preventive value in UK PCE needs to be tested with such challenges. More research is needed to understand whether and how these values are given weight in real-life decision- making about cognitive enhancement drugs. It may be that, rather than explicit measures to control PCE, these implicit values can form the basis of ‘nudge’ techniques to promote UK students’ resilience to PCE in institutional settings where use is thought to be causing harm.

The relatively small, qualitative sample in our study necessarily limits the generalizability of our findings. Still, our work adds to a body of quantitative and qualitative research that shows with progressive clarity that access to cognitive enhancement drugs is another important mediator of risk and of resilience to PCE use in university settings. Student access to PCE is contingent, in part, on the regulation of prescription drugs; therefore, regulatory pathways that mediate prescription drug access offer an obvious mechanism for managing PCE in universities.

Varying levels of PCE have been reported (largely anecdotally) across UK universities. One way to better understand how the value of competition works, both to promote PCE and to inhibit it, would be to examine PCE prevalence and practices among universities of different rankings in the UK, and among different subject courses. In addition, though we are wary of ascribing generalizing characteristics to national populations, we are curious if values around competition and national stereotypes might contribute to further understanding the differences in PCE prevalence across universities in different countries.

## Conclusion

Forlini and Hall [9] argue that the gap between ethical theories and empirical evidence must be bridged with research on neuroenhancement that studies individual values within a social infrastructure. Our focus group study suggests a moral ecology that operates within the social infrastructure of the university. The architecture of this ecology consists of collective values, social dynamics and individual factors that shape PCE attitudes and practices within institutional contexts. Consequently the moral ecology should be viewed as incorporating key mechanisms of PCE risk and resilience. Our study suggests that in UK universities, PCE risk and resilience is mediated by collective values around competition and prescription drug taking, and soft peer pressure and social norms within friendship groups.

Our work also suggests that access to PCE does not determine risk and resilience in a straightforward way. The influence that access to means of PCE exerts on risk and resilience outcomes is likely to be moderated by social and cultural factors, such as the collective values, social dynamics and group norms illuminated in this study. More evidence about the moral ecologies of PCE will enable proportionate and targeted policy responses and better institutional decision-making about PCE education and intervention.

## Footnotes

1 The Russell Group is comprised of 24 universities, which are generally ranked among the top universities in the United Kingdom.
